# Progress in Fevers

**Published:** 1901-04-06

**Authors:** 


### PROGRESS IN FEVERS.

**Typhoid.**—F. J. Smith[1] considers the stools and appetite are the prime factors in determining treatment. At least one stool should be carefully examined daily for undigested food, blood and sloughs. These indicate revised diet and caution. Blood in large quantity demands starvation and free exhibition of opium. Appetite measures the healthy digesting power of the stomach, and, in the absence of vomiting, hæmorrhage or distension, may be satisfied by eggs, bread and milk, milk puddings, pounded fish or meat in small quantities, besides fluids. Complete loss of appetite, on the other hand, demands complete withholding of food. Plain water alone may advantageously be given even for four or five days. Tympanites, a grave symptom, best prevented by judicious diet, should be watched for daily and treated by 2 drams of sodium sulphate in warm water every two hours till the bowels act freely. Purdy[2] advocates digestible solid food: the tongue not the fever is the guide. W. M. Brown,[3] A. H. Smith[4] and Billings[5] also favour abolition of routine milk diet. Osler[6] pleads for operation in cases of perforation, which form 25 per cent. of typhoid mortality. He has thus saved 30 to 40 per cent. of such cases. Diagnosis and operation must be *immediate*. Sudden pain, intense and paroxysmal, with change in abdominal signs and consistency are the chief indications. The shock of operation is surprisingly well borne. W. Taylor[7] also favours early laparotomy.

Official statistics[8] show the results of inoculation by Professor Wright's method among the 15th Hussars in India. Incidence among the inoculated was 0·55 per cent., mortality 0·27 per cent.; among the uninoculated incidence was 6·14 per cent., mortality 3·35 per cent. Of 57 persons of the *personnel* of the Scottish National Red Cross Hospital twice inoculated, Cayley[9] reports none took the disease, and their blood gave good reactions to Widal's test four months later. J. W. Smith[10] describes the constitutional effects as sudden, with severe prostration for two days, fever, rigors, local effusion and œdema, and swelling of glands. Tooth[11] considers whirlwinds, dust, and flies largely responsible for the South African outbreak. Flies show special predilection for enteric patients. An outbreak among soldiers at Quetta[12] suggests infection by dust from infected latrines.

Parkes and Rideal[13] record experiments to provide a practicable and portable water disinfectant. They find 15 grains of acid sodium bisulphate sterilises 1 pint of typhoid-infected water in 15 minutes. Sufficient 5-grain tabloids to last a soldier on the march three weeks could be packed into a ¼-lb. tin.

F. W. Clarke[14] suggests that the apparent immunity of Asiatics is due to protection by an attack in infancy. Maclagan[15] ascribes the symptoms of typhoid to the enormous multiplication of bacilli in the tissues. Fever is due to increased tissue activity, wasting and thirst to the appropriation by swarms of hungry young bacilli of the nitrogen and water intended for the tissues. When the nidus in the intestinal glands is exhausted fever ceases. Recrudescence is due to destruction of fresh glands and renewed multiplication. Relapse is recrudescence deferred.

Smith[16] found marked tenderness of calf muscles very common at No. 9 General Hospital, and also "tender toes" an extremely painful condition, probably a neuritis, occurring during convalescence from typhoid. Ogilvie[17] reports several cases of jaundice apparently dependent on the typhoid process, and Marsden[18] records a case of typhoid with ulceration and perforation of the gall bladder. A pseudo-typhoid bacillus coli was obtained from the gall bladder cultures. Nicholls and Learmonth[19] record at length a case of typhoid with multiple hæmorrhages. The fatty degeneration of the various endothelia and pavement membranes found may offer some explanation of the hæmorrhages.

[1] Lancet, Feb. 2. [2] New Zealand Med. Jour., Nov. [3] Med. Rec., Dec. 1. [4] and [5] Amer. Jour. Med. Sc., Nov. [6] Lancet, Feb. 9. [7] Dub. Jour. Med. Sc., Jan. 1. [8] Lancet, Feb. 9. [9] Brit. Med. Jour., Jan. 12. [10] Med. Chron., Jan. [11] Brit. Med. Jour., Nov. 10. [12] Ibid., p. 1291. [13] Lancet, Jan. 26. [14] Brit. Med. Jour., Jan. 26. [15] Lancet, Jan. 12. [16] Med. Chron., Jan. [17] Brit. Med. Jour., Jan. 12. [18] Med. Chron., Jan. [19] Lancet, Feb. 2.

